# The Molecular Allergen Recognition Profile in China as Basis for Allergen-Specific Immunotherapy

**DOI:** 10.3389/fimmu.2021.719573

**Published:** 2021-08-27

**Authors:** Nishelle D’souza, Milena Weber, Eszter Sarzsinszky, Susanne Vrtala, Mirela Curin, Mirjam Schaar, Victoria Garib, Margarete Focke-Tejkl, Yanqiu Li, Richard Jones, Hao Chen, Rudolf Valenta, Baoqing Sun

**Affiliations:** ^1^Division of Immunopathology, Department of Pathophysiology and Allergy Research, Center for Pathophysiology, Infectiology and Immunology, Medical University of Vienna, Vienna, Austria; ^2^Worg Pharmaceuticals, Hangzhou, China; ^3^Department of Allergy and Clinical Immunology, State Key Laboratory of Respiratory Disease, National Clinical Research Center for Respiratory Disease, Guangzhou Institute of Respiratory Health, First Affiliated Hospital of Guangzhou Medical University, Guangzhou, China; ^4^Laboratory of Immunopathology, Department of Clinical Immunology and Allergology, Sechenov First Moscow State Medical University, Moscow, Russia; ^5^National Research Center (NRC) Institute of Immunology Federal Medico-Biological Agency (FMBA) of Russia, Moscow, Russia; ^6^Karl Landsteiner University of Health Sciences, Krems, Austria

**Keywords:** allergy, molecular diagnosis, IgE, AIT = allergen-specific immunotherapy, allergen, allergy vaccine

## Abstract

Approximately 30% of the world population suffers from immunoglobulin-E (IgE)-mediated allergy. IgE-mediated allergy affects the respiratory tract, the skin and the gastrointestinal tract and may lead to life-threatening acute systemic manifestations such as anaphylactic shock. The symptoms of allergy are mediated by IgE-recognition of causative allergen molecules from different allergen sources. Today, molecular allergy diagnosis allows determining the disease-causing allergens to develop allergen-specific concepts for prevention and treatment of allergy. Allergen-specific preventive and therapeutic strategies include allergen avoidance, vaccination, and tolerance induction. The implementation of these preventive and therapeutic strategies requires a detailed knowledge of the relevant allergen molecules affecting a given population. China is the world´s most populous country with around 1.4 billion inhabitants and an estimated number of more than 400 million allergic patients. Research in allergy in China has dramatically increased in the last decade. We summarize in this review article what is known about the dominating allergen sources and allergen molecules in China and what further investigations could be performed to draw a molecular map of IgE sensitization for China as a basis for the implementation of systematic and rational allergen-specific preventive and therapeutic strategies to combat allergic diseases in this country.

## Introduction

### Allergy and Its Evolution in Childhood

Immunoglobulin E (IgE) – associated allergy is the most frequent immunologically mediated hypersensitivity disease affecting more than 30% of the world´s population. Allergic patients suffer from a variety of clinical symptoms which include hay fever (i.e., rhinitis, conjunctivitis), asthma, skin inflammation (e.g., urticaria, atopic dermatitis), gastrointestinal allergy (e.g., oral allergy syndrome-OAS, vomiting, diarrhea) and life-threatening systemic anaphylactic shock (1). Of note, allergic patients may suffer from isolated allergic symptoms in certain organs (i.e., monomorbidity) or from various allergic co-morbidities affecting several different organs at the same time and thus present with different allergic phenotypes which may also vary over time (2). The detailed analysis of the evolution of allergic sensitization from early childhood to adolescence suggests that allergic sensitizations occur early in life and, depending on genetic and environmental factors, in particular in response to repeated allergen contact, progress from clinically silent forms of IgE sensitizations which are characterized by the presence of low levels of allergen-specific IgE antibodies in the blood without accompanying symptoms towards symptomatic allergy (3, 4). The analysis of molecular IgE sensitization profiles in birth cohorts has shown that asymptomatic IgE sensitization frequently precedes symptomatic allergy which then starts with mild symptoms such as allergic rhinitis and consecutively progresses to severe forms such as allergic asthma (5–7). Therefore, allergy may be compared with major diseases affecting mankind such as cancer, cardiovascular diseases, autoimmune diseases, metabolic/endocrine diseases (e.g., Diabetes) which start in a clinically silent form which can only be detected by preventive medical examination through laboratory analyses in phenotypically still “healthy” subjects.

### The Importance of Early Allergen-Specific Prevention Taking Local Sensitization Profiles Into Account

Similar as for all the aforementioned non-communicable diseases which heavily affect mankind and create a huge burden to the health care systems, early preventive intervention strategies are needed for allergy. Only the introduction of preventive measures and in particular of early vaccination holds the promise that they will lead to more effective treatment and thus save huge health care costs. In the case of allergy, preventive strategies include mainly allergen-specific forms of prevention, i.e., allergen avoidance and allergy vaccination, i.e., allergen-specific immunotherapy, AIT (8–12). Regarding the detection of early asymptomatic IgE sensitization in childhood and the consecutive development of allergic manifestations much has been learned in birth cohort studies conducted in a European Union-funded research program (Mechanisms of the Development of Allergy MeDALL) (1). A particular strength of the MeDALL project was that it enabled to study the molecular IgE sensitization profiles in birth cohorts with the MeDALL chip which contained a large number of micro-arrayed allergen molecules (13). This allowed to study the evolution of molecular IgE sensitization from early childhood to adolescence in the longitudinal studies and to understand the variations of regional IgE sensitization profiles in cross-sectional studies involving patients from different regions of a particular country. For example, it could be demonstrated that molecular IgE sensitization profiles are determined by the exposome (i.e., the occurrence of particular allergen sources in certain regions) demonstrating that climate conditions and certain habits in a population have effects on allergen exposure and consequently on allergen sensitization profiles in the population. One of the early studies performed with recombinant birch pollen allergens in different regions of Europe reported strikingly different frequencies of IgE sensitizations to the major birch pollen allergen Bet v 1 and cross-reactive birch pollen allergens from North to South-Europe (14) and variations of weed allergen-specific IgE sensitization profiles were reported for different European regions and the United States (15). A study conducted in different regions of France exemplified the dependence of molecular IgE sensitization profiles on climate conditions (16). Another study analyzing the molecular IgE sensitization profile of allergic patients from the Philippines demonstrated that symptomatic allergy was caused mainly by house dust mite and pet allergens whereas grass pollen-specific IgE sensitization was mainly due to IgE to carbohydrate epitope-bearing allergens such as Phl p 4 and Cyn d 1 which did not cause allergic symptoms (17). In fact, IgE sensitization to carbohydrate epitopes usually does not lead to clinical symptoms of allergy (18–20).

Interestingly, changes of the exposome by anthropologic as well as natural changes in the biome may have effects on IgE sensitization profiles in a population already from one to another generation (21).

### China as Model for Allergen-Specific Prevention

**Figure 1:**
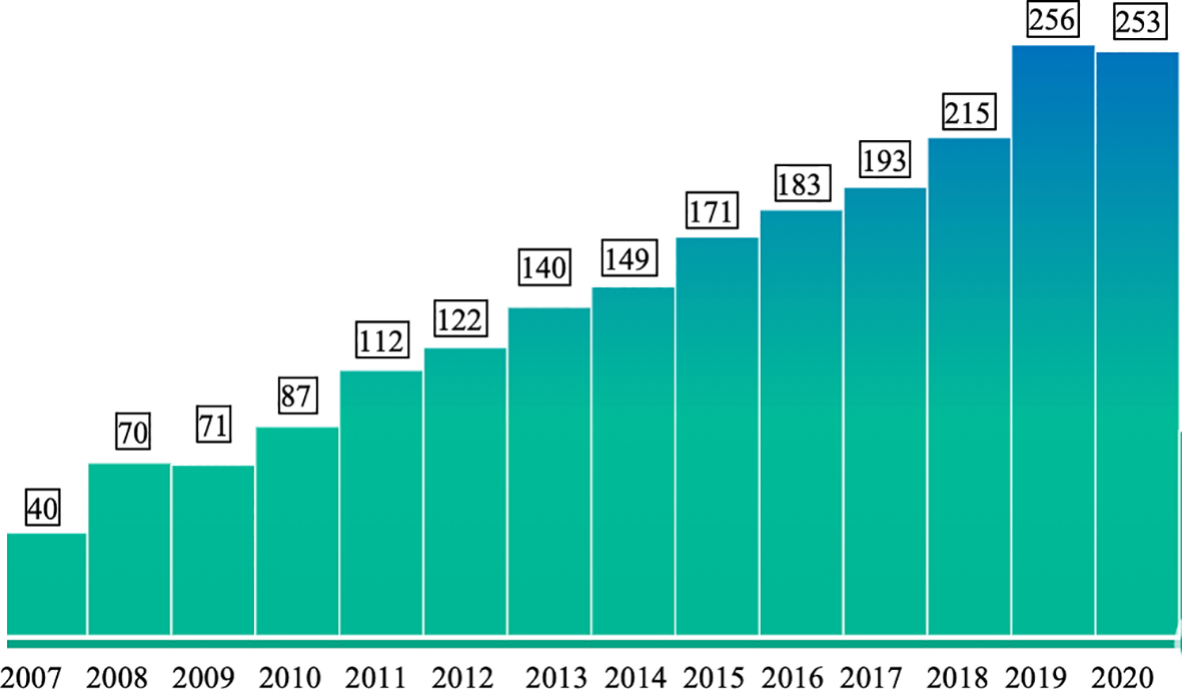
Numbers of publications per year (y-axis) from 2007 to 2020 (x-axis) which can be retrieved from PubMed (https://pubmed.ncbi.nlm.nih.gov/) with the keywords “Allergy” and “Allergen” and “China”.

**Figure 2:**
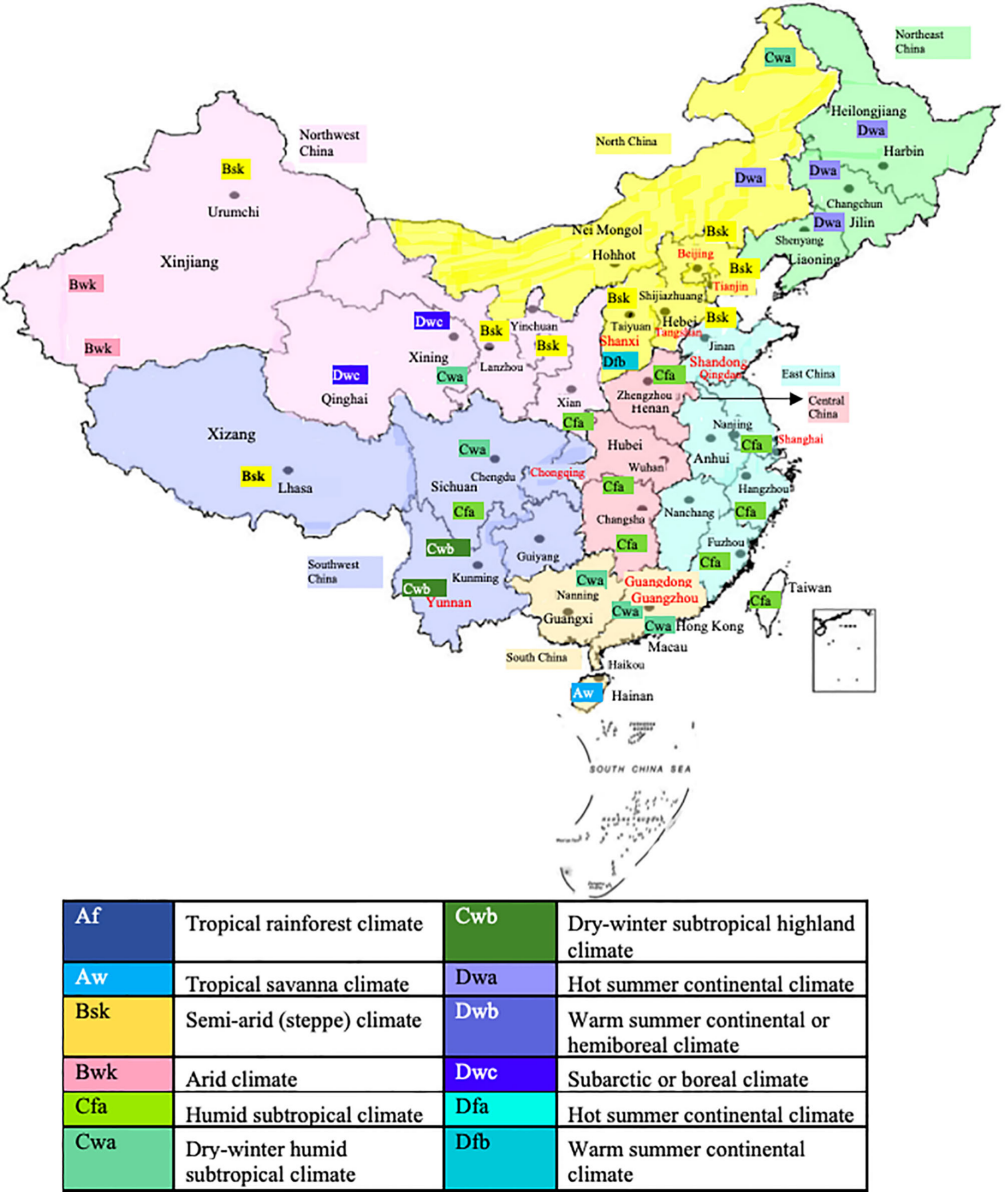
Territories inhabited mainly by a Chinese population and cities/areas for which allergen sensitization data have been obtained. Climate conditions according to the Köppen-Geiger climate classification system are indicated.

In China, the country with the largest population of around 1.4 billion, allergy research has made rapid progress in the last decade. **Figure 1** shows the numbers of publications which can be retrieved from PubMed under the terms “Allergy”, “Allergen” and “China” from the years 2007 to 2020. The annual number of publications including the three keywords increased from 40 to more than 250 in this period indicating the interest in the disease-causing allergens. In fact, the territory inhabited by a mainly Chinese population covers an area of approximately 9.6 million square kilometers and is the second-largest country in the world by land area after Russia (**Figure 2**). The territory inhabited by the Chinese population lies between latitudes 18° and 54° North and longitudes 73° and 135° East comprising a vast and diverse landscape from the Gobi and Taklamakan Desert in the arid north to the subtropical forests in the wet south. We have indicated the diversity of the different climate areas of the territory inhabited by the Chinese population in **Figure 2** according to the Köppen-Geiger climate classification system which is summarized in **Table 1** (22, 23). The different climate types are indicated by a combination of a first letter indicating tropical, arid, temperate, continental, or polar climate and additional one or two letters as indicated in **Table 1**. Due to the occurrence of many different climate areas, China has over 34.000 species of animals and vascular plants making it the third-most biodiverse country in the world. Accordingly, one must assume that many different allergen sources and allergen molecules thereof are responsible for allergic sensitizations in different parts of the country.

**Table 1:** Climate classification according to the Köppen-Geiger climate classification system.

1^st^ letter	2^nd^ letter	3^rd^ letter
A (Tropical)	f (Rainforest)	
	m (Monsoon)	
	w (Savanna, Dry winter)	
	s (Savanna, Dry summer)	
B (Arid)	w (Desert)	
	f (Steppe)	
		H (hot)
		K (Cold)
C (Temperate)	w (Dry winter)	
	f (No dry season)	
	s (Dry summer)	
		a (Hot summer)
		b (Warm summer)
		c (Cold summer)
D (Continental)	w (Dry winter)	
	f (No dry season)	
	s (Dry summer)	
		a (Hot summer)
		b (Warm summer)
		c (Cold summer)
		d (Very cold winter)
E (Polar)	T (Tundra)	
	F (Eternal frost (ice cap))	

The specification of the climate conditions includes the first, second and third letter as indicated.

In order to understand what is known regarding the role of different allergen sources and allergen molecules in the territory inhabited by the Chinese population we have reviewed scientific studies performed in this area to analyze allergic sensitization profiles.

## Frequencies of Allergic Sensitizations to Allergen Sources in China: First Information From Allergen Extract-Based Studies

In the last ten years several studies have been performed to identify relevant allergen sources in the Chinese area. We have summarized in **Table 2** studies performed by skin testing with allergen extracts, IgE serology using allergen extracts or by questionnaires (24–36). **Table 2** indicates the regions where the studies had been performed, the climate prevailing in the region, what subjects (e.g., general population, patients with allergic symptoms, etc.) were tested, what type of tests had been used (sIgE serology, skin prick testing, questionnaire) and what was considered a positive test result. Importantly, we list what types of allergen extracts had been tested for a better understanding of the prevalence of sensitizations to given allergen extracts in the study populations. In this context it is important to understand that frequencies of detected sensitizations can only be compared for the allergen extracts which have been tested in the different studies and regions because the studies vary considerably regarding the selection of tested allergen extracts. Furthermore, it is important to understand that allergen extract-based testing cannot discriminate between genuine sensitization to the given allergen source and between co- and cross-sensitization (37, 38). This information can only be obtained by molecular testing (39–41). It should be also noted that both, skin prick testing (SPT) and IgE serology can only detect sensitization, but the sensitization does not necessarily need to be linked to clinical symptoms. Positive SPT results and sIgE serology results may also occur in subjects without allergic symptoms (42). Yet there are fundamental differences between IgE serology and SPT. The measurement of specific IgE clearly demonstrates that a subject mounts specific IgE antibodies and thus unambiguously confirms the condition of “IgE sensitization”. However, certain “allergens” may bind specifically IgE antibodies but do not induce cross-linking of effector cell-bound IgE and hence do not cause inflammatory reactions. A good example for such “IgE-reactive antigens” without allergenic activity are carbohydrate-containing determinants also termed CCDs which are present as cross-reactive moieties in a large number of unrelated allergen extracts and hence may give “false-positive” IgE test results meaning that for example a subject shows CCD-specific IgE reactivity but no allergic reaction upon contact with the allergen source (38, 43, 44). In contrast to IgE serology, SPT measures if an allergen extract can induce effector cell (i.e., mast cell, basophil) activation and thus informs if the sensitized subject may experience an allergic reaction to the tested extract. However, whether indeed an allergic reaction by natural allergen exposure occurs cannot be determined by SPT because this may depend on the extent of allergen exposure, epithelial barrier function, levels and clonality of the IgE response to name a few factors influencing allergen-induced inflammation (45, 46). The fact, that SPT and IgE serology are not always predicting allergic reactions needs special consideration in food allergy because some food allergens are easily degraded upon uptake in the gastrointestinal tract whereas others are more resistant to degradation, and this cannot be determined by these tests but may require provocation testing such as food provocation (47). Major limitations of allergen extract-based diagnostics are that they do not allow identification of the culprit allergen molecules and that due to poor quality of natural allergen extracts, false positive and negative results can occur (48). All of the aforementioned considerations need to be taken into account when it comes to the interpretation of studies shown in **Table 2**.

**Table 2:** Allergen-specific IgE sensitization in China according to questionnaire or allergen extract-based diagnosis.

Region	Climate according to the Köppen-Geiger climate classification system	Number of subjects	Type of test	Definition of results	Allergen sources tested and percentage of positive subjects for each tested source	Reference
Yunnan, Southwest China	Cfb Temperate, no dry season, warm summer	1431 subjects with suspected allergy	Allergen-extract-based serology, quantitative, ImmunoCap	sIgE levels ≥0.35 IU/mL were defined as positive.	Allergen sources tested: d1: house dust mite, *Dermatophagoides pteronyssinus*), cockroach (i6, *Blatella germanica*), tree pollen mix (tx4: *Quercus alba*, *Ulmus americana*, *Platanus acerifolia*, *Salix caprea*, *Populus deltoides*), mold mix (mx1: *Penicillium chrysogenum*, *Cladosporium herbarum*, *Aspergillus fumigatus*, *Alternaria alternata*), dog dander (e5), crab (f23), shrimp (f24), egg white (f1), and milk (f2)	Zeng et al. (24)
					In total 60.9% were sIgE positive	
					Frequencies of detected sensitizations were HDM: 44.9%	
					Cockroach: 26.5%	
					Tree pollen mix: 21.6%	
					Mold mix: 4.3%	
					Dog dander: 5.9%	
					Crab: 20.8%	
					Shrimp: 18.7%	
					Egg white: 13.4%	
					Milk: 8.5%	
Guangdong, South China	Cfa	1347 subjects with suspected allergy	Allergen-extract-based serology, quantitative, ImmunoCap	sIgE levels ≥0.35 IU/mL were defined as positive.	Allergen sources tested:	Zeng et al. (24)
	Temperate, no dry season, hot summer				d1: house dust mite, *Dermatophagoides pteronyssinus*), cockroach (i6, *Blatella germanica*), tree pollen mix (tx4: *Quercus alba*, *Ulmus americana*, *Platanus acerifolia*, *Salix caprea*, *Populus deltoides*), mold mix (mx1: *Penicillium chrysogenum*, *Cladosporium herbarum*, *Aspergillus fumigatus*, *Alternaria alternata*), dog dander (e5), crab (f23), shrimp (f24), egg white (f1), and milk (f2)	
					In total 57.8% were sIgE positive	
					HDM: 43.3%	
					Cockroach: 20.9%	
					Tree pollen mix: 5.3%	
					Mold mix: 3.6%	
					Dog dander: 4.5%	
					Crab: 16.3%	
					Shrimp: 17.4%	
					Egg white: 13.4%	
					Milk: 14.8%	
Guangzhou, South China	Cfa Temperate, no dry season, hot summer	1497 allergic patients	Allergen-extract-based serology, quantitative, ImmunoCap	sIgE levels ≥0.35 IU/mL were defined as positive.	Allergen sources tested: Der p, Der f, and Blo t	Liu et al. (25)
					Results: Der p: 85.50%	
					Der f: 85.37%	
					Blo t: 71.54%	
					Co-sensitized: 70.14%	
Various allergy centers, China	Several	6304 allergic patients	Skin prick testing with allergen extracts SPT Allergen-extract-based serology, quantitative, ImmunoCap IgE inhibition studies in 1236 sera	Guideline-based skin prick testing for Der p, American and German cockroach	Der p: 88%	Sun et al. (26)
					Per a: 25.7%	
					Bla g: 18.7%	
Southern China	Cfa Temperate, no dry season, hot summer	39813 allergic patients	Allergen-extract-based serology, quantitative, ImmunoCap	sIgE levels ≥0.35 IU/mL were defined as positive.	d1: house dust mite, *Dermatophagoides pteronyssinus*), cockroach (i6, *Blatella germanica*), crab (f23), egg white (f1), and milk (f2)	Luo et al. (27)
					Results: HDM: 28.1%	
					Cockroach: 24.3%	
					Shrimp: 19.2%	
					Crab: 15.5%	
					Egg white: 9.9%	
					Milk: 9.6%	
Yunnan, Southwest China	Cfb Temperate, no dry season, warm summer	7759 allergic patients	Allergen-extract-based serology, quantitative, ImmunoCap	sIgE levels ≥0.35 IU/mL were defined as positive.	Allergen sources tested: d1: house dust mite, *Dermatophagoides pteronyssinus*), cockroach (i6, *Blatella germanica*), mold mix (mx1: *Penicillium chrysogenum*, *Cladosporium herbarum*, *Aspergillus fumigatus*, *Alternaria alternata*), dog dander (e5), crab (f23), shrimp (f24), egg white (f1), and milk (f2).	Luo et al. (28)
					In total 45.6% were sIgE positive	
					Results: Cockroach: 27.0%	
					HDM: 25.6%	
					Mold mix: 3.9%	
					Dog dander: 3.3%	
					Shrimp: 18.8%	
					Crab: 15.6%	
					Egg white: 9.5%	
					Milk: 7.4%	
Southern China	Cfa	1839 children with respiratory diseases and cow´s milk sIgE	Allergen-extract-based serology, quantitative, ImmunoCap	sIgE levels ≥0.35 IU/mL were defined as positive.	Allergens tested: Cow´s milk extract.	Huang et al. (29)
	Temperate, no dry season, hot summer				Natural alpha lactalbumin (ALA), beta lactoglobulin (BLA), casein (CAS) were tested in 103 children:	
					Results: Cow’s milk: 36.7%	
					ALA: 87.4%	
					BLG: 86.4%	
					CAS: 69.9%	
Shanghai, East China	Cfa	7996 allergic patients	Skin prick testing with allergen extracts SPT	A wheal size of ≥ 3 mm, after subtracting the negative control value was considered positive.	Allergen extracts and control solutions were supplied by ALK (Horsholm, Denmark).	Yan et al. (30)
	Temperate, no dry season, hot summer				Results: *Dermatophagoides farinae*: 73.10%	
					*Dermatophagoides pteronyssinus*: 72.21% *Blomia tropicalis*: 53.10%	
					*Blattella germanica*: 31.18%	
					*Periplaneta americana*: 27.75%	
					dog dander: 24.96%	
					mixed molds: 17.56%, shrimp: 17.02%	
Chongqing, Southwest China	Cwa	142 allergic children	Allergen-extract-based serology, quantitative, ImmunoCap	sIgE levels ≥0.35 IU/mL were defined as positive.	Allergen extracts tested: Der p, Der f, Blo t, cat dander, dog dander, egg white, milk, cockroach, shrimp, and crab.	Zeng et al. (31)
	Temperate, dry winter, hot summer				Results: Der p: 100%	
					Der f: 100%	
					Blo t: 91.84%,	
					Cat: 10.96%,	
					Dog: 7.32%,	
					Egg white: 9.15%,	
					Milk: 11.58%,	
					Cockroach: 17.03%,	
					Shrimp: 18.90%,	
					Crab: 18.28%	
Guangdong, South China	Cfa	2540 subjects	Questionnaire	Positive answer checked by clinician	Total food allergy prevalence rate 4%	Zeng et al. (32)
	Temperate, no dry season, hot summer					
					Detailed results: shrimp: 4.4%	
					crab: 3.2%	
					mango: 2.3%	
					cow’s milk and dairy products: 1.9%	
					eggs: 1.4%	
Shanghai, East China	Cfa	3479 asthmatic children	Skin prick testing with allergen extracts SPT	Positive SPT result not defined	Allergen sources tested: HDM, cat fur, dog fur, ragweed pollen, willow pollen, shrimp, crab, egg, milk, cashew nut:	Mao et al. (33)
	Temperate, no dry season, hot summer					
					HDM: 51.0%	
					cat: 33.2%	
					dog: approx. 30%	
					ragweed: 37.7%	
					willow pollen: approx. 18%	
					shrimp and crab: approx. 7%	
					egg, milk, and cashew nut: each less than 5%	
Qingdao, East China	Cwa	2841 allergic children	Retrospective analysis of Skin prick testing with allergen extracts SPT	Ratio of allergen wheal to histamine wheal of >26% was considered positive	Allergen extracts tested: Der p, Der f, *Penicillium notatum*, *Aspergillus fumigatus*, late spring flower, summer autumn flower, weed, ragweed, latex, *Gramineae*, shrimp, mussel, carp, milk, egg, peanut, peach, eel, hemp, sea crab	Lin et al. (34)
	Temperate, dry winter, hot summer				Results obtained for children older than 6 years:	
					Der p: 72.0%	
					Der f: 70.8%	
					*Penicillium notatum*: 42%	
					*Aspergillus fumigatus*: 34.3%	
					late spring flower: 39.6%	
					summer autumn flower: 39.6%	
					weeds: 25.3%	
					latex: 28.3%	
					*Gramineae*: 28%	
					mussel: 38.7%%	
					shrimp: 38.6%	
					carp: 39%	
					milk: 24.8%	
					egg: 22.1%	
					peach: 20.8%	
					eel 5.7%	
					hemp: 5.4%	
					sea crab: 2.1%	
Multicenter	Four regions, North, East, Western south, Southern coast **Figure 3**, different climates	6304 patients suffering from asthma and/or rhinitis according to questionnaire	Skin prick testing with allergen extracts	Wheal size ≥ 3mm after subtraction of negative control	Allergen extracts tested: Der p, Der f, Blo t, dog, cat, cockroach (American and German), *Artemisia vulgaris*, *Ambrosia artemisifolia*, mixed grasses, mixed trees, mold mix I and IV, ALK (Horsholm, Denmark).	Sun et al. (26)
					Results for all regions: Der f: 59%, Der p: 57.6%, Blo t: 40.7%, American cockroach: 16.1%, dog: 14%, German cockroach: 11.5%, *Artemisia vulgaris*: 11.3%, cat: 10.3%, *Ambrosia artemisifolia*: 6.5%, mixed mold I: 6.3%, mixed mold IV: 4.4%, mixed grass pollen: 3.5%, mixed tree pollen: 2.2%	
					Results for each region separately in **Figure 3**.	
Multicenter, Mainland China	Several regions Northwest, North, Northeast, Southwest, Central, East, South, different climates	44156 patients with allergic symptoms	Allergen-extract-based serology, quantitative, ImmunoCap	sIgE levels ≥0.35 IU/mL were defined as positive.	Allergen extracts tested: House dust mite (d1), German cockroach (i6), tree pollen mix (tx4), mold mix (mx1), dog dander (e5), egg white (f1), cow´s milk (f2), crab (f23), shrimp (f24)	Luo et al. (35)
					Results: House dust mite: 33.74%, cockroach: 24.5%, shrimp: 19.97%, crab: 17.31%, cow’s milk: 11.62%, egg white: 10.92%	
					tree pollen mix- 9.35% dog dander- 4.02%, mold mix: 3.92%	
					Results for each region in **Figure 4**.	
Eastern Taiwan area	Cfa	15455 allergic patients (children and adults) seen between 2010-2015	Allergen-extract-based serology, quantitative, ImmunoCap	sIgE levels >0.70 IU/mL were defined as positive.	Allergen extracts tested: Der f, Der p, cow´s milk, egg white, shrimp, cockroach, crab, dog, wheat, ragweed, Candida albicans, yeast, cat, peanut, soybean, rice, pork, melon, codfish, egg yolk, Aspergillus fumigatus, A. tenuis, orange, Pencillium notatum.	Chen et al. (36)
	Temperate, no dry season, hot summer				Results adults (>18 years; approximately): Der f: 53%; Der p: 52%; cow´s milk: 5%; egg white: 3%; shrimp: 32%; cockroach: 25%; crab: 31%; dog: 22%; wheat: 9%; ragweed: 8%; Candida albicans: 18%; yeast: 6%; cat: 8%; peanut: 5%; soybean: 9%; rice: 3%; pork: 5%; melon: 3%; codfish: 2%; egg yolk: <1%; Aspergillus fumigatus: 6%; Alternaria alternata: 2%; orange: <1%; Pencillium notatum: 3%; Results children (< 18 years, approximately: Der f: 80%; Der p: 78%; cow´s milk: 32%; egg white: 32%; shrimp: 23%; cockroach: 22%; crab: 20%; dog: 18%; wheat: 15%; ragweed: 10%; Candida albicans: 9%; yeast: 8%; cat: 7%; peanut: 6%; soy bean: 5%; rice: 3%; pork: 3%; melon: 2.5%; codfish: 2%; egg yolk: 2%; Aspergillus fumigatus: 2%; Alternaria alternata: 1%; orange: 1%; Pencillium notatum: <1%.	

Since the importance of allergen sources may strongly vary depending on climate conditions, we have visualized the centers and the corresponding climate in the region in which the studies had been performed in **Figure 2** and in **Table 2**.

**Figure 3:**
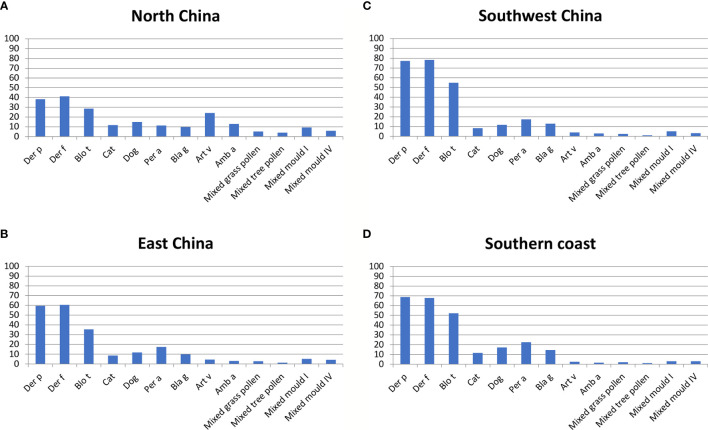
Frequencies (y-axes: percentages of positive subjects) of allergic sensitizations to different allergen extracts according to (26) (x-axes: Der p, Der f, Blo t, cat, dog, Per a, Bla g, Art v, Amb a, mixed grass pollen, mixed tree pollen, mixed mold I and mixed mold IV) as determined by skin prick testing in different areas of China **(A)**: North China; **(B)**: East China; **(C)**: Southwest China; **(D)**: Southern Coast). Depending on the tested region, frequencies of IgE sensitization varied approximately as follows: Der p: 39-78%; Der f: 40-79%; Blo t: 28-52%; cat: 8-12%; dog: 12-18%; Per a: 10-22%; Bla g: 10-12%; Art v: 3-25%; Amb a: 2-12%; mixed grass pollen: 2-7%; mixed tree pollen: 1-4%; mixed mold I: 3-10%; mold IV: 3-7%.

Various studies on allergen sources were found for several regions of China of which the most surveyed are Yunnan and Guangdong (**Figure 2**, **Table 2**) (24, 25) These tropical regions of China inhabit over 150 million of the Chinese population and their unique topography and climate play a great role in the allergens detected in these areas. According to the studies performed, HDM, cockroach and tropical/sub-tropical mites (i.e., *Blomia tropicalis*) as well as dog and cat represent important respiratory allergen sources in China. Regarding sensitization to pollen and mold allergens there seemed to be considerable variation of sensitization rates. Studies from southern regions of China showed low sensitization rates to pollen and molds of less than 10% (24, 27) whereas a considerable number of pollen and mold-sensitized subjects >20% was found in central (33) and eastern parts (34) of China (**Table 2**). Two multicenter studies should be mentioned because they have analyzed different regions of China and thus allow comparing sensitization rates to the same panel of tested allergen sources in different regions. A study by Sun et al. (26) has investigated 6304 allergic patients from different regions of China by skin testing with a broad panel allergen extract from respiratory allergen sources (**Table 2**). We have compared the prevalence of IgE sensitization determined in different regions of China according to the latter study (26) in **Figure 3**. Results obtained demonstrate that the frequencies of HDM, *Blomia tropicalis* and cockroach sensitization are lowest in North China (**Figure 3A**) and highest in East and especially South China (**Figures 3B–D**) By contrast, pollen sensitizations especially to mugwort and ragweed and likewise mold sensitizations were highest in North China (**Figure 3A**) but lowest in South China (**Figure 3C, D**). Interestingly the rates of sensitization to dog and cat were comparable in each of the investigated regions (**Figures 3A–D**). Although the study by Sun et al. (26) was a very large study it does not cover the complete territory inhabited by the Chinese population. Furthermore, sensitization rates may vary by age and may be different in children versus adults.

**Figure 4:**
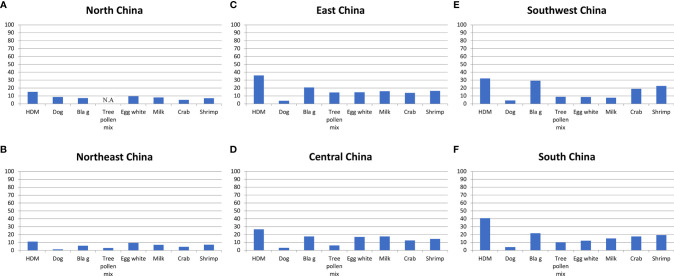
Frequencies (y-axes: percentages of positive subjects) of IgE sensitizations to different allergen extracts according to (35) (x-axes: HDM; dog; Bla g; tree pollen mix; egg white; milk; crab; shrimp) as determined by IgE serology in different areas of China (**(A)**: North China; **(B)**: Northeast China; **(C)**: East China; **(D)**: Central China; **(E)**: Southwest China; **(F)**: South China). Depending on the tested region, frequencies of IgE recognition varied approximately as follows: HDM: 11-40%; dog: 1-9%; Bla g: 7-30%; tree pollen mix: 3-14%; egg white: 8-17%; milk: 7-18%; crab: 4-19%; shrimp: 8-22%.

Another multicenter study was performed by IgE serology in 44156 patients with allergic symptoms by Luo et al. (35) (**Table 2**). However, this study has tested a different panel of allergen extracts. Instead of weed and grass pollen, only a tree pollen allergen mix was tested, *Blomia tropicalis* was not included but extracts from milk, crab and shrimp were part of the tested allergen extract panel (**Table 2**; **Figure 4**). Nevertheless, this study by Luo et al. (35) conducted in patients with allergic symptoms confirmed the findings of the earlier mentioned SPT study by showing that HDM and cockroach sensitization rates were much lower in Northern China than in Southern China highlighting the North-to-South gradients even more clearly by including data from Central China (**Figures 4A–F**). Again, the rates of dog sensitization were quite comparable in each of the studied regions, and this was also found for milk sensitization (**Figures 4A–F**). When comparing sensitization rates determined by SPT and IgE serology we found a good concordance of results for the regions regarding the prevalence of sensitization but we also noted some discrepancies between results. For example, the rates of sensitization determined by SPT for HDM were much higher than those found by IgE serology. These discrepancies may be explained by differences regarding the tested patients, differences in the composition of the allergen extracts used and differences regarding sensitivity and specificity of the used methods (SPT versus IgE serology).

Of note, the rates of sensitization to crab and shrimp were lowest in those regions where HDM and cockroach sensitization was low (i.e., North and Northeast China) and increased towards South China (**Figures 4A–F**). The latter observation is quite interesting because it is quite possible that IgE reactivity with cross-reactive allergens found in HDM and cockroach such as tropomyosin and arginine kinase and crab and shrimp may be responsible for the association of the IgE responses between the respiratory and food allergen sources. Accordingly, it was of interest to review data regarding molecular IgE sensitization profiles from China to understand the fine specificities of IgE sensitization in greater detail.

## Information Regarding Molecular Sensitization Profiles in China Through First Studies Performed With Molecular Diagnostics

When reviewing allergen extracts based studies performed with allergen extracts in China, we noted already that many comprehensive studies including large numbers of subjects had been performed. Most of the studies mentioned in **Table 2** enrolled more than 1000 subjects but were limited to serology and therefore further investigations taking clinical parameters are needed. Studies performed in such large numbers of patients have only been performed in certain birth cohort studies in Europe but are lacking for most other parts of the world (49). Likewise, we found that also a considerable number of studies had been performed to analyze the molecular IgE sensitization profiles of patients from different regions of China with purified natural and recombinant allergen molecules (**Table 3**). Specific IgE reactivity to purified natural and recombinant respiratory and food allergens was analyzed in different regions of China (**Table 3**) (50–63).

**Table 3:** IgE sensitization to allergen molecules determined in different regions of China by IgE serology.

Region	Climate according to the Köppen–Geiger climate classification system	Number of subjects	Type of test	Definition of results	Allergen molecules tested and percentage of positive subjects for each tested allergen	Reference
Guangdong, South China	Cfa	432 mite allergic patients	EUROIMMUN system (EUROIMMUN, Oumeng Medical Laboratory, Lübeck, Germany)	≥0.35 IU/mL were defined as sIgE-pos- itive.	Allergens tested: Der f 1, Der f 2, Der p 1, Der p 2, Der p 3, Der p 5, Der p 7, Der p 10, Der p 23, Blo t 5, Pen a 1, Eri s 1, Bla g 1, Bla g 2, Bla g 4, Bla g 5, Per a 7.	Huang et al. (50)
	Temperate, no dry season, hot summer				Results: Der f 1: 74.07%, Der f 2: 77.78%, Der p 1: 81.48%, Der p 2: 66.20%, Der p 3: 1.85%, Der p 5: 15.05%, Der p 7: 29.40%, Der p 10: 10.65%, Der p 23: 54.63%, Blo t 5: 28.01%, Pen a 1: 4.17%, Eri s 1: 10.42%, Bla g 1: 0.23%, Bla g 2: 9.03%, Bla g 4: 1.16%, Bla g 5: 5.56%, Per a 7: 4.40%.	
Guangzhou, South China	Cfa	57 polysensitized mite allergic patients	ImmunoCap Immuno-Solid phase Allergy Chip (ISAC)	sIgE levels ≥ 0.30 ISU were classified as positive.	112 allergen molecules of the ImmunoCAP Immuno-Solid phase Allergy Chip (ISAC) (Thermo Fisher Scientific, Uppsala, Sweden), Results: nDer f 1: 71.9%, rDer f 2: 73.7%, nDer p 1: 70.1%; rDer p 2: 66.7%; rDer p 10: 10%, rBlo t 5: 10%, rLep d 2: 10%; rFel d 1: 29.8%, rCan f 1: 14%, nPhl p 4: 12.3%, nCyn d 1: 17.5%, CCD: 7.0%, nPen m 1: 8.8%, nJug r 2: 8.8%, nPen m 2: 5.3%, rAsp f 3: 8.8%, rAsp f 1: 7.0%, rAlt a 1: 5.3%, rAsp f 6: 5.3%, rAlt a 1: 3.5%, nBla g 7: 8.8%, rAni s 3: 8.8%. n	Hu et al. (51)
	Temperate, no dry season, hot summer					
Guangzhou, South China	Cfb	258 allergic patients of whom 58 who were positive for Bermuda, timothy and Humulus scandens were tested with allergen molecules	Allergen-extract-based and allergen component-based serology, quantitative, ImmunoCap	≥0.35 IU/mL were defined as sIgE-pos- itive.	Allergens tested in 35 timothy-positive subjects: Phl p 4, Phl p 1, Phl p 5, Phl p 6, Phl p 7, Phl p 11, Phl p 12.	Luo et al. (52)
	Temperate, no dry season, Warm summer				Results: Phl p 4: 100%, Phl p 1: 17.1%, Phl p 5: 8.6%, Phl p 6: 8.6%, Phl p 7: 8.6%, Phl p 11: 8.6%, Phl p 12: 8.6%.	
Guangdong South China	Cfb	268 HDM allergic patients who	Euroimmun system (Euroline;	≥0.35 IU/mL were defined as sIgE positive.	Positive results with allergen extracts in the population: Dog (e1): 54.85%, cat (e2): 63.81%, cow (e4): 13.06%, sheep (e81: 10.45%, rat (e73): 10.82%	Chen et al. (36)
	Temperate, no dry season, warm summer	were SPT positive for cat and/or dog	EUROIMMUN, Lubeck, Germany)		Allergens tested: Can f 1, Can f 2, Can f 3, Can f 4, Can f 5, Fel d 1, Fel d 2.	
					Results: Can f 1: 17.54%, Can f 2: 7.46%, Can f 3: 7.46%, Can f 4: 7.84%, Can f 5: 9.70%, Fel d 1: 61.19%, Fel d 2: 11.19%	
Shanxi and Shandong provinces, North China and Yunan province, Southwestern China	Dwa Continental, dry winter, hot summer Cwb Dry-winter subtropical highland climate	240 allergic patients	Allergen-extract-based serology, quantitative, ImmunoCap	≥0.35 IU/mL were defined as sIgE positive.	Allergens tested: nArt v 1, nArt ar 2, nArt v 3, nArt an 7 plus mugwort pollen extract	Gao et al. (53)
					Results: nArt v 1: 84%, median sIgE: 9.6kUA/L, nArt ar 2: 48%, median sIgE 0.2 kUA/L, nArt v 3: 66%, median sIgE 1.1kUA/L, nArt an 7: 87%, median sIgE 2.1kUA/L	
					mugwort pollen: 100%, median sIgE 30.4 kUA/L	
Guangzhou, South China	Cfb	78 patients sensitized to Bermuda grass	Allergen- component-based serology, quantitative, ImmunoCap	≥0.35 IU/mL were defined as sIgE positive.	Allergens tested: Cyn d 1 (g216), Cyn d 12, Phl p 1 (g205), Phl p 4 (g208), Phl p 5 (g215), Phl p 7 (g210), Phl p 12 (g212), Art v 1 (w231), Art v 3 (w233), Art v 4 (w234).	Liao et al. (54)
	Temperate, no dry season, Warm summer				Results: Cyn d 1 (g216): 24.4%, Cyn d 12: approx. 8%, Phl p 1 (g205): 12.8%, Phl p 4 (g208): 7.7%, Phl p 5 (g215): approx. 3%, Phl p 7 (g210): approx. 5%, Phl p 12 (g212): 9.0%, Art v 1 (w231): approx. 6%, Art v 3 (w233): 10.3%, Art v 4 (w234): 10.3%.	
Southern China	Cfa	200 sIgE Der p-positive patients with allergic asthma and/or rhinitis	Allergen- component-based serology, quantitative, ImmunoCap ImmunoCap Immuno-Solid phase Allergy Chip (ISAC)	≥0.35 IU/mL were defined as sIgE positive for quantitative ImmunoCap and ≥0.30 ISU were defined as sIgE positive for ISAC.	All 200 were tested for IgE to Der p 1, Der p 2 and Der p 10 by quantitative ImmunoCap and 75 were tested by ISAC	Zeng et al. (55)
	Temperate, no dry season, hot summer				Results: Der p 1 and/or Der p 2: 91.5%, Der p 10: 6%. Der p 10 positives showed also IgE reactivity to other tropomyosins: Ani s 3: 67%, Bla g 7: 67%, Pen m 1: 75.3%.	
Northern China	Dwa Continental, dry winter, hot summer	48 patients with milk allergy	IgE ELISA	Mean optical density values plus two standard deviations measured for non-cow´s milk allergic subjects were considered as cut-off for a positive result	Natural purified cow´s milk allergens from Sigma-Aldrich Co: α-lactalbumin (Bos d 4), ß-lactoglobulin (Bos d 5), α-casein (Bos d 9), ß-casein (Bos d 11), κ-casein (Bos d 12).	Li et al. (56)
					Results: α-lactalbumin (Bos d 4): 22.9%, ß-lactoglobulin (Bos d 5): 50%, α-casein (Bos d 9): 41.7%, ß-casein (Bos d 11): 56.3%, κ-casein (Bos d 12): 50%.	
Tianjin, China	Dwa	56 egg allergic children	Light-initiated chemiluniscent assay (LICA)	Mean relative light units plus two standard deviations measured for non-cow´s milk allergic subjects were considered as cut-off for a positive result	Natural purified egg allergens from Sigma-Aldrich Co: nGal d 1, nGal d 2, nGal d 3, nGal d 4, nGal d 5.	Zhang et al. (57)
	Continental, dry winter, hot summer				Results: nGal d 1: 51.8%, nGal d 2: 62.5%, nGal d 3: 41.1%, nGal d 4: 14.3%, nGal d 5: 23.2%.	
Beijing, China	Dwa	402 pollinosis patients	Allergen- component-based serology, quantitative, ImmunoCap	≥0.35 IU/mL were defined as sIgE positive	Allergens tested: rBet v 1, rBet v 2, Art v 1, Art v 3, Pru p 1, Pru p 3.	Li et al. (58)
	Continental, dry winter, hot summer				Results: 85% (n=31) of birch pollen allergic patients (n=37) were positive for Bet v 1. Art v 1 was more prevalent (i.e., 79%) in pollen-food allergic patients than in pollen allergic patients without food allergy (i.e., 33%). IgE reactivities to Bet v 1 and Pru p 1 and between Art v 3 and Pru p 3 were significantly correlated.	
Beijing, China	Dwa	148 mugwort allergic patients of whom 107 suffered also from plant food allergy	Allergen- component-based serology, quantitative, ImmunoCap	≥0.35 IU/mL were defined as sIgE positive	Allergens tested: Art v 1, Art v 3, Pru p 3, Pru p 1, Pru p 4, Ara h 9, Cor a 8.	Deng and Yin (59)
	Continental, dry winter, hot summer				Results Art v 1: 81.1%, Art v 3: 73.0%, Pru p 3: 90% of peach allergic patients (n=88), Ara h 9: 88% of peanut allergic patients (n=17), Cor a 8: 80% of hazelnut allergic patients (n=15).	
Northern China, Tangshan	Dwa	203 allergic patients, sera collected between February and July 2014	Allergen component based serology, quantitative, ADVIA Centaur and ImmunoCap	≥0.35 IU/mL were defined as sIgE positive	Allergen extracts and allergens tested: Birch pollen extract, Bet v 1, Mal d 1, Gly m 4	Hao et al. (60)
	Continental, dry winter, hot summer				Results obtained: Birch pollen positive sera: 16.7% (n=34) of which 82.4% were Bet v 1 positive. 96.4% and 78.6% of Bet v 1-positive samples were positive for Mal d 1 and Gly m 4, respectively.	
Beijing, China	Dwa	83 pollen food allergic patients (PFS) and 46 only pollen allergic patients (PS)	Allergen- component-based serology, quantitative, ImmunoCap	≥0.35 IU/mL were defined as sIgE positive	Pollen allergen sources positive in more than 50% of patients: White ash, poplar,birch, Juniper, elm, phoenix tree, mugwort, Humulus japonicas, willow, ragweed, goosefoot, Cirsium jaonicum, Kochia scoparia, Bermuda grass, timothy grass. Allergens tested and with results: Bet v 1, Art v 3, Gly m 4, Pru p 1, Pru p 3.	Ma et al. (61)
	Continental, dry winter, hot summer				Results: Bet v 1: PFS: 37.3%; PS: 19.6%) Art v 3: PFS: 62.7%; PS: 34.8%, Gly m 4: PFS: 28.9%; PS: 10.9%, Pru p 1: PFS: 31.3%; PS: 13.0%, Pru p 3: PFS: 57.8%; PS: 34.8%.	
Northern China	Dwa	70 mugwort allergic patients 24 allergic to peach and mugwort 15 only peach 31 only mugwort	Allergen- component-based serology, quantitative, ImmunoCap	≥0.35 IU/mL were defined as sIgE positive	Allergens tested: rPru p 1, rPru p 3, rPru p 4, nArt v 1, nArt v 3	Gao et al. (62)
	Continental, dry winter, hot summer				Results: Larger part of mugwort-peach allergic patients had higher IgE to Art v 3 and lower to Pru p 3. Few had only Pru p 3-specific IgE.	
Guangzhou city, China	Cfb	18 patients with bronchopulmonary aspergillosis (ABPA) and 54 Aspergillus-sensitized asthma patients (Af)	Allergen- component-based serology, quantitative, ImmunoCap	≥0.35 IU/mL were defined as sIgE positive	Allergens tested: Asp f 1, Asp f 2, Asp f 3, Asp f 4, Asp f 6	Luo et al. (63)
	Temperate, no dry season, Warm summer				Results (ABPA, Af): Asp f 1: 88.89% *vs*. 59.26%, Asp f 2: 66.67% *vs*. 33.33%, Asp f 3: approx. 66% *vs*. 50%, Asp f 4: 61.11% *vs*. 33.33%, Asp f 6: 66.67% *vs*. 14.81%.	

The study by Huang et al. (50), provides interesting information about IgE sensitizations to HDM, *Blomia tropicalis* and cockroach allergens (**Table 3**). This study confirms the importance of the major HDM allergens, Der p 1, Der p 2 and Der p 23 in a population from South China and shows that Der p 7 and Der p 5 are also quite relevant molecules. Interestingly, the rate of sensitization to Blo t 5 (28%) was considerably higher than to the related allergen, Der p 5 (15%) from HDM indicating that *Blomia tropicalis* may be an independent and important allergen source whereas frequencies of IgE reactivity to Der p and Der f allergens were almost identical. IgE sensitization rates to cockroach allergen molecules were low (<10%) in the HDM population analyzed by Huang et al. The frequencies of sensitization and importance of Der p 1 and Der p 2 were confirmed in other studies (**Table 3**) (51, 55). Evidence for IgE cross-reactivity between HDM tropomyosin, Der p 10 and tropomyosins from other invertebrates including food was found by Zeng et al. (55) indicating Der p 10 as the genuinely sensitizing allergen.

The impact of *Blomia tropicalis* allergens is highly seen in children and there are various data and evidence that show the health implications of this particular allergen source. While the study performed by Kidon et al. (64), was based in Singapore, over 75% of the study population were of Chinese origin. Kidon et al. (64) reported 70% incidence of Blo t allergen sensitization among 253 students and the most prominent allergen molecules were Blo t 5, Blo t 7, Blo t 15 and Blo t 21. The health implications of this allergy can highly vary: some patients have fewer symptoms while others may have upper and lower respiratory tract problems with skin manifestations. Kidon et al. suggest that this may be due to the patient’s qualitative allergic sensitization/responses to perennial allergens in their surroundings. A comparison of sensitization to *Blomia* and *Dermatophagoides* in Singapore and China showed large differences and suggests Blo t 5 as marker allergen for accurate discrimination of IgE sensitization to *Blomia* and *Dermatophagoides* (64). Accordingly, the sensitization pattern for Chinese people may be dominated by their living environment (65).

The Singapore and China comparison also shows a high reactivity of subjects in China to amylase (Blo t 4) which would be indicative cross reactivity with anti-scabies. China has one of the highest burdens of scabies infections in the world which will have affected at least 20% of the population and (66) therefore molecular surveys should include binding to the group 4 for current and past infections and group 20 for current infections (67).

Quantitative measurements by ImmunoCAP or micro arrays eventually with basophil activation experiments performed with allergen molecules and not only prevalence data would be critical to determine the allergic load and thus the reagents needed for optimal immunotherapy. For optimal immunotherapy allergen molecules with clinical relevance as reflected by high IgE binding capacity and allergenic activity need to be identified (43).

The molecular analyses summarized in **Table 3** provided further interesting insights. For example, in certain parts of southern China grass pollen sensitization seemed to be due to cross-reactive carbohydrates because most grass pollen sensitized patients mainly reacted with nPhl p 4 and nCyn d 1 whereas only few showed IgE reactivity to the clinically relevant grass pollen allergens such as Phl p 1, Phl p 5, Phl p 6, Phl p 12 (52, 54). Additional studies have been performed in the western and northern parts of China, but results are not yet available. The importance of mugwort pollen allergy was highlighted by several studies identifying Art v 1 and Art v 3 as the most important allergens in terms of frequencies of IgE recognition and allergen-specific IgE levels (53, 59). Art an 7 was proposed as an important mugwort allergen but the levels of specific IgE were much lower than those specific for Art v 1 and no recombinant allergen without glycosylation is yet available to inform about the importance of peptide versus carbohydrate epitopes in its IgE recognition (53). In addition, the importance of cat and dog sensitizations was underlined by the molecular studies identifying especially Fel d 1 and to a lower extent of Can f 1 as important allergens (36). Another interesting finding was that birch pollen allergy as confirmed by IgE reactivity to the major birch pollen allergen, Bet v 1 was found in patients from Beijing and Northern China (60). Bet v 1 patients also suffered from oral allergy syndrome due to cross-reactivity with Bet v 1-related PR10 allergens from plant food (60). Thus, the possible importance of birch pollen allergy should not be neglected in China, especially in the northern and central regions. In this context it should be mentioned that IgE sensitization to Art v 3, the LTP from mugwort seemed to be also responsible for plant-derived food allergy due to IgE cross-reactivity with the LTP from peach, Pru p 3 (62). In addition, IgE sensitization to *Humulus lupulus* and profilin thereof seems to be important in China (68).

A comprehensive study performed with recombinant *Aspergillus fumigatus* allergens confirmed the importance of using these molecules for diagnosis of bronchopulmonary aspergillosis and Aspergillus-related asthma (63). Furthermore, similar IgE recognition profiles for milk and egg allergen molecules were reported in China (56, 57) as compared to milk- and egg-sensitized populations from other countries (69, 70).

## Open Questions Which Need to be Addressed by Molecular Diagnosis

**Figure 5:**
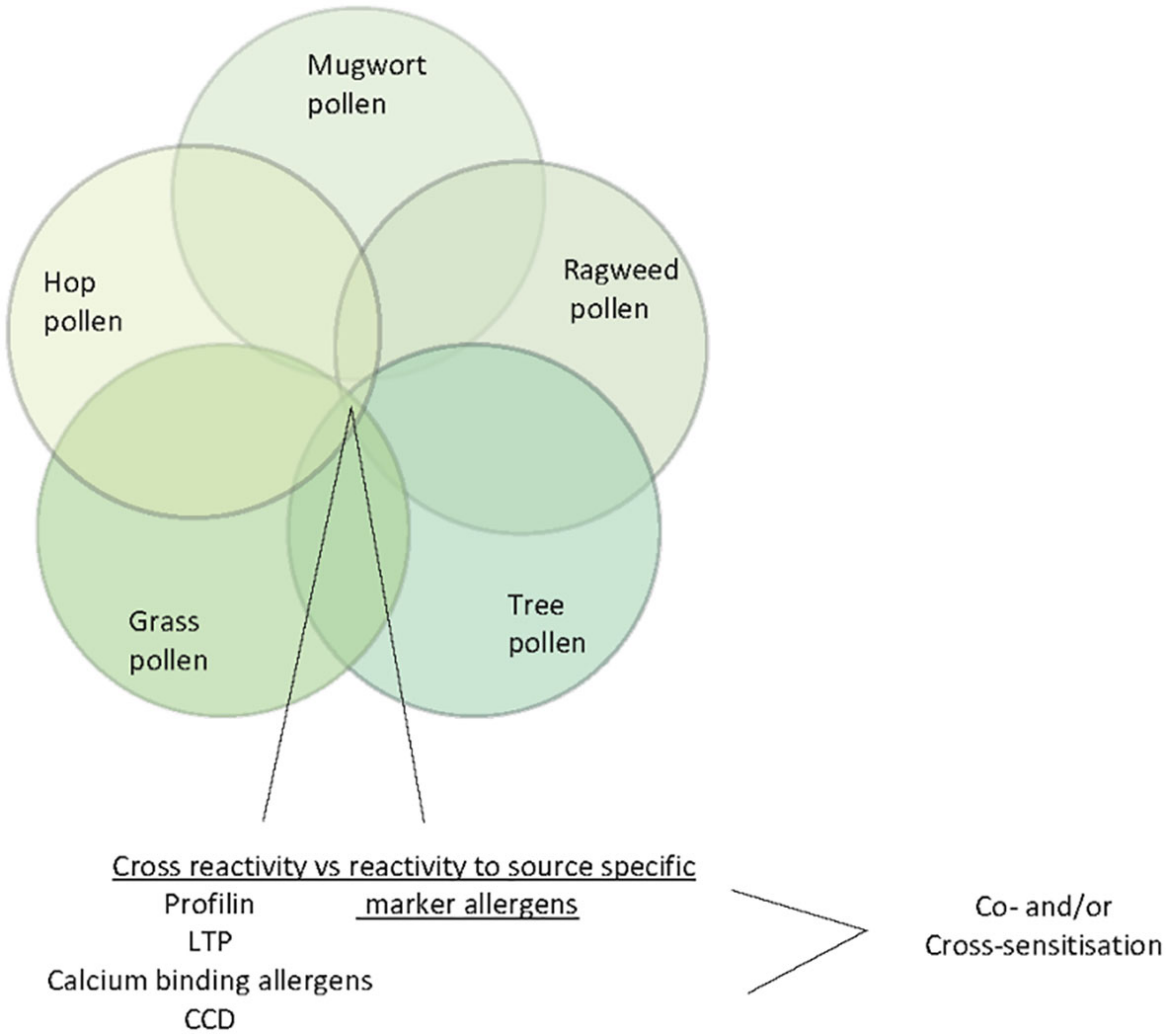
Venn diagram of pollen allergen sources (ragweed, mugwort, tree pollen, hop pollen and grass pollen) indicating cross-reactive allergens/IgE-reactive antigens (profilin, LTP, calcium-binding allergens and CCDs).

It is well established that the identification of the genuine-sensitizing allergen sources with allergen extracts is hampered by the presence of cross-reactive allergens in different allergen sources (41). Patients exhibiting IgE reactivity to such cross-reactive allergens may show allergic symptoms and IgE reactivity to different, often unrelated allergen sources containing such cross-reactive allergens. Studies performed 30 years ago have shown that structural and/or sequence similarity among cross-reactive allergens is the basis for IgE cross-reactivity (71, 72) Profilins which are ubiquitous actin-binding proteins occurring in eukaryotic organisms were identified as cross-reactive allergens especially in pollen of plants and plant-derived food (72, 73). Likewise, lipid transfer proteins have been identified as allergens in plant-derived food and also pollen but due to more limited sequence similarity, IgE cross-reactivity is less pronounced (74, 75). Calcium-binding proteins containing different numbers of calcium-binding sites have been identified in pollen of unrelated plants as highly cross-reactive allergens (76–78). While the aforementioned cross-reactive plant allergens show allergenic activity and are thus responsible for clinical symptoms, carbohydrate-containing determinants (CCDs) show extensive IgE cross-reactivity but little or no allergenic activity (79, 80). CCDs occur in many different unrelated allergen sources (e.g., plants, insects, venoms, house dust mites, molds etc.) as cross-reactive IgE epitopes. Accordingly, CCD-reactive allergic patients may show IgE cross-reactivity to these unrelated allergen sources and be falsely considered to be polysensitized (20). The studies carried out in China with allergen extracts indicate sensitization to pollen from trees, weeds, grasses, and other plants but diagnosis based on allergen extracts can be confounded by the presences of cross-reactive allergens such as profilin, calcium-binding allergens, LTPs and CCDs in pollen from different plants (**Figure 5**). Only molecular diagnosis will allow to clarify if a patient is co- or cross-sensitized and help identifying the most important allergen sources.

Allergic sensitization to animal dander allergens can also be confounded by the presence of cross-reactive allergens in different animals. For example, albumin has been identified as a cross-reactive allergen in many animals and cow´s milk (81, 82). IgE cross-reactivity between Fel d 7 and Can f 1 may confound the differential diagnosis of cat and dog allergy (83) and Fel d 4-related allergens may occur as cross-reactive allergen in different animals (84). Accordingly, molecular diagnosis will be necessary to determine the correct frequency of allergic sensitization to different animals and to identify the culprit allergen source responsible for sensitization.

**Figure 6:**
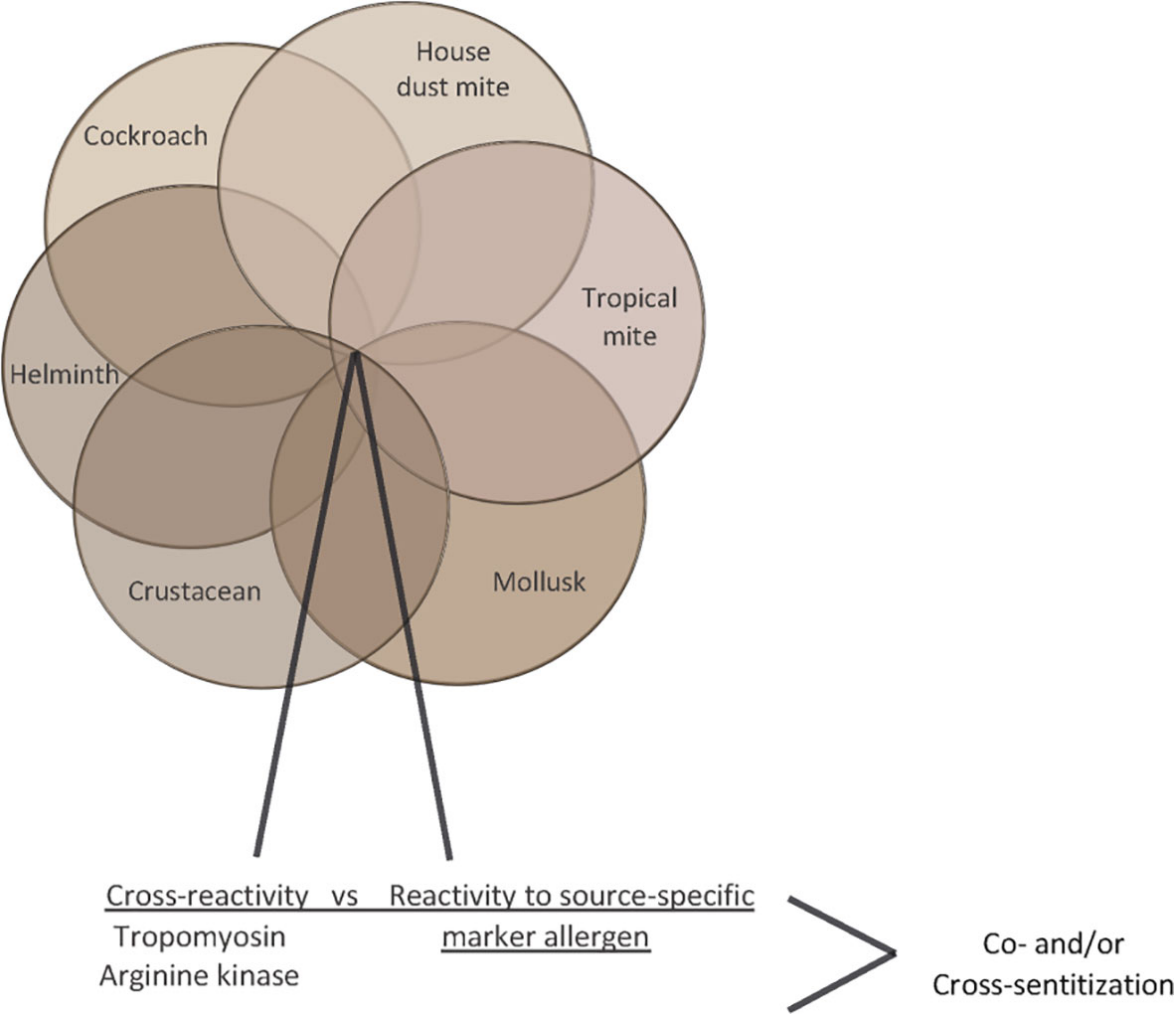
Venn diagram of invertebrate allergen sources (house dust mites, tropical mites, cockroach, crab, shrimp) indicating cross-reactive allergens (tropomyosin, arginine kinase).

Likewise, diagnosis of allergic sensitization to HDMs, tropical mites (e.g., *Blomia tropicalis*), cockroach, crab, shrimp, and other seafood belonging to the invertebrates can be obscured by IgE reactivity to cross-reactive allergens such as tropomyosin and arginine kinase whereas glutathione S transferase may serve as source-specific marker allergen due to low cross-reactivity of GST cockroach, dust mites and helminths (**Figure 6**) (85–90). In this context we noted that IgE sensitization and skin sensitivity to shrimp and crab were especially common in areas of China where HDM sensitization rates were high (**Table 2**).

**Figure 7:**
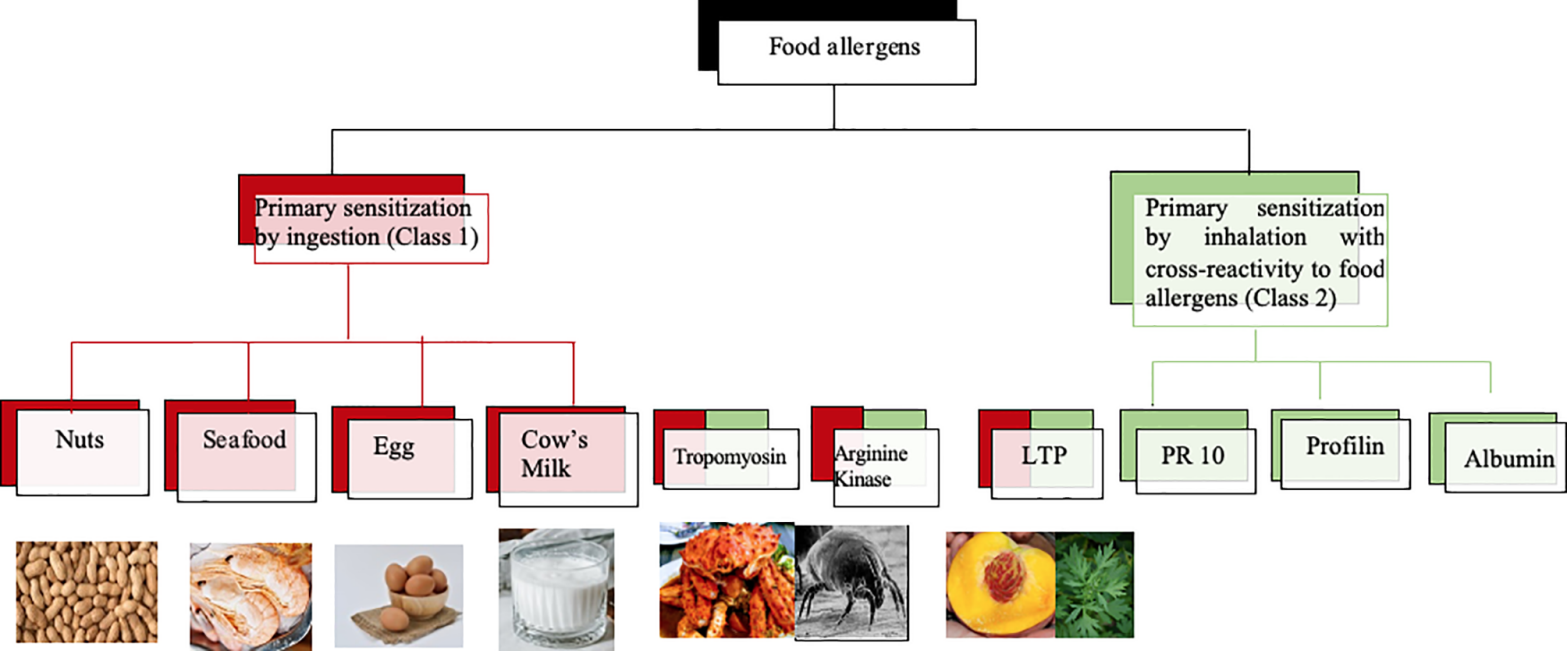
Class 1 and class 2 food allergens/allergen sources with possible relevance for Chinese patients. Class 1 food allergens/allergen sources are indicated in red, class 2 food allergens in green whereas allergens which may behave as class 1 and 2 allergens contain red and green color.

Molecular diagnosis has also become of high importance in the diagnosis of food allergy (47). IgE recognition of class 1 food allergens which usually sensitize *via* ingestion and represent stable proteins which are poorly degraded in the gastrointestinal tract is usually related with severe and systemic symptoms of food allergy. By contrast, class 2 food allergens are proteins which sensitize *via* the respiratory tract and with exceptions (e.g., LTPs which can serve also as class 1 food allergens) they are usually easily degraded in the gastrointestinal tract and hence cause mainly mild and not life-threatening symptoms of allergy restricted to the mouth (e.g., oral allergy syndrome) or to chronic allergic inflammation (e.g., atopic dermatitis) in other organs caused by activation of T cell epitope containing peptides without anaphylactic activity. **Figure 7** provides an overview of some food allergen sources containing class 1 food allergens and mentions food allergen molecules which can serve as class 2 (e.g., PR10 allergens, profilin, albumin) and in certain cases also as class 2 and/or as class 1 food allergens (e.g., LTP, tropomyosin, arginine kinase). The fact, that food allergen sources can contain class 1 and class 2 allergens at the same time emphasizes the importance of molecular diagnosis for the prediction of the likelihood of a patient to experience no, mild, or severe forms of food allergy symptoms. In addition, it is important to understand if a respiratory or food allergen source was responsible for sensitization. Several epidemiological research studies on allergen molecules have been performed in China but the detection of molecular IgE sensitizations responsible for clinically meaningful cross-reactivity will need confirmation by case history, IgE-inhibition studies, and provocation testing.

## Towards a Complete Map of the Molecular IgE Sensitization Profile in China as a Basis for Allergen-Specific Prevention and Treatment

**Figure 8:**
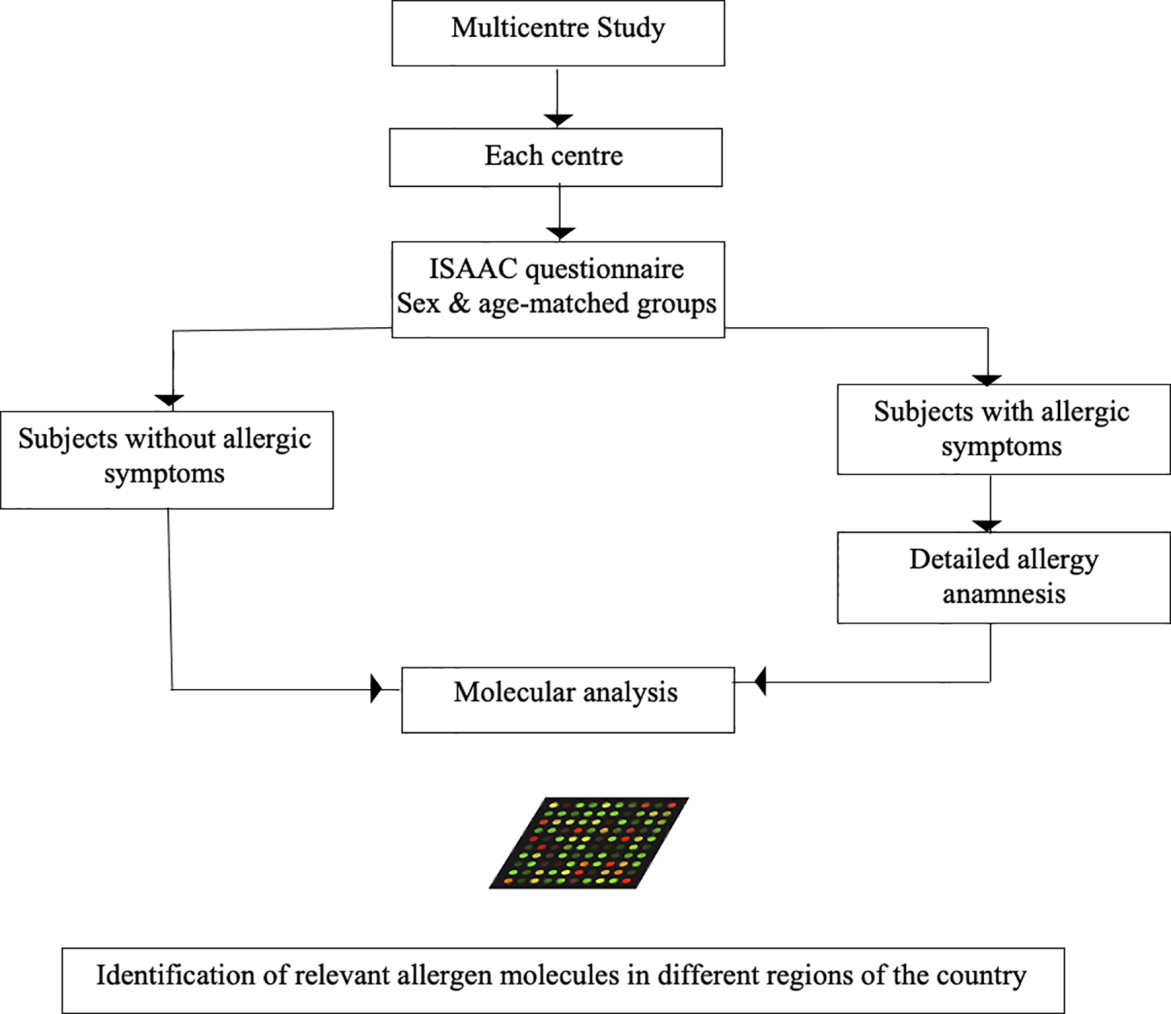
Possible design of a multicenter study to determine the relevant allergen molecules in the different regions of China. Sex- and age matched groups of equal number with or without allergic symptoms according to ISAAC questionnaire are obtained from the regional population of each center. In subjects with allergic symptoms a detailed anamnesis of allergic symptoms is performed. Sera from the subjects with and without allergic symptoms are then analyzed in an anonymized and blinded manner to determine the molecular IgE sensitization profiles in a qualitative (i.e., nature of IgE-positive allergens) and quantitative (i.e., allergen-specific IgE levels) manner to define the relevant allergen molecules in each region of the country.

The identification of the clinically most relevant allergen molecules is a prerequisite for the rationale establishment of allergen-specific strategies for treatment and prevention. The unbiased testing of IgE sensitization to a large panel of allergen molecules has become possible with the generation of chips containing micro-arrayed allergen molecules (40). Such chips allow the testing of IgE reactivity to hundreds of purified allergen molecules with only microliter volumes of serum. However, molecular testing needs to be compared with allergen extract based testing and sensitivity and specificity should be determined especially in patients with low IgE sensitization. The MeDALL allergen chip which has been created within an EU-funded research program (Mechanisms of the Development of Allergy MeDALL) (1) is an example for such a chip which has been successfully used to determine molecular IgE sensitization profiles in European birth cohorts (13). A similar, but advanced technology (91) could be used to create allergen microarrays for the assessment of IgE sensitization profiles in different regions of China. The advantage of testing a large panel of allergen molecules by a discovery approach instead of testing with a pre-selected limited panel of allergen molecules or allergen extracts is that the multiallergen screening test approach is not biased, precisely identifies the allergen molecules and thus allows to discover yet unanticipated sensitizations. As explained above, it is important to link IgE recognition profiles and allergen-specific IgE antibody levels with clinical symptoms in order to identify clinically relevant sensitizations to particular allergens and/or groups of allergens. In this context it should be mentioned that several studies show that not only specific IgE levels to a particular allergen but also cumulative specific IgE levels to several allergen molecules are associated with types and severity of symptoms and allergic phenotypes (2, 92, 93). Multiallergen diagnosis seems to be particularly suitable for linking IgE-sensitization profiles with disease phenotypes. Regarding China, it seems that an approach of testing subjects from different regions of the country will be needed to differentiate the IgE sensitization profiles in the different regions because data available from allergen extract-based testing have already indicated that IgE sensitization profiles will vary between different geographic regions depending on the local exposomes (**Tables 2**, **3**; **Figures 3** and **4**). We know from birth cohort studies that IgE-reactivity profiles consolidate between 10-16 years of age (6, 7) and are then stable in adult patients (94). Accordingly, it is suggested to perform a cross-sectional multicenter study involving different regions and to enroll adolescent subjects who were born and raised in the investigated area to appropriately grasp the local sensitization profiles. In a recent study conducted in Moscow, Russia (4), it turned out to be of advantage to investigate not only subjects with allergic symptoms but to also investigate a sex- and age-matched control group of subjects without allergic symptoms. This study design actually allowed to identify subjects with a clinically-silent IgE sensitization which allows to characterize the features of a clinically silent IgE sensitization and to compare it with IgE sensitization profiles of subjects with symptoms. This study design has enabled to identify clinically irrelevant types of allergens (e.g., CCDs), IgE reactivity profiles and/or specific IgE threshold levels associated with symptoms and certain phenotypes of disease. For example, CCDs on Phl p 4 and Cyn d 1 were identified as clinically irrelevant “allergens” (17, 95) and IgE threshold levels to major birch pollen allergen, Bet v 1, associated with symptomatic birch pollen allergy and certain phenotypes of birch pollen allergy (i.e., oral allergy syndrome) were found (93). Ideally, subjects for a cross-sectional study should not yet have undergone allergy diagnosis because inclusion of patients from allergy centers focusing on certain forms of allergy (e.g., respiratory allergy, food allergy or skin manifestations) may create unwanted selection bias. Therefore, it is recommended to recruit subjects *de novo* for a cross-sectional survey by advertisement and to use an established questionnaire such as the ISAAC questionnaire (96), to form equally sized and matched groups of subjects with and without symptoms of allergy (**Figure 8**). Based on an anticipated frequency of allergy of 30% and IgE sensitizations of up to 60% it should be sufficient to analyze approximately 100-200 subjects with and 100-200 subjects without allergy symptoms in each of the centers to obtain an overview of IgE sensitization profiles. A detailed allergy anamnesis according to a procedure which is identical for each of the study centers is recommended for subjects with allergy symptoms to clinically define allergic phenotypes in the group of subjects with allergic symptoms. It may well be that not only IgE sensitization profiles, but also allergic phenotypes may vary in different regions. For example, seafood allergy may be more prominent in regions where seafood is consumed, and seasonal allergy may be more frequent in the regions with heavy pollen exposure.

Serum samples obtained in the different study centers should then be anonymized and analyzed in a blinded manner in a core laboratory to allow unbiased analysis and to minimize or avoid variations regarding the sIgE measurements due to methodological issues caused by different equipment and variation of test batches. Results from the multiallergen testing and clinical parameters can then be pooled in a database and be subjected to further analysis to identify clinically relevant allergens for each region as a basis for rational allergen-specific treatment and preventive strategies (10). In addition, a wealth of additional data may be expected which can be implemented in diagnostic and predictive algorithms useful for precision medicine treatment (97, 98).

## Summary and Conclusions

China is the country with the world´s largest population comprising approximately 1.4 million with an estimated number of at least 400 million allergic patients. Accordingly, the development of affordable allergen-specific diagnosis, treatment and prevention strategies for allergy is a major health priority in this country. The basis for allergen-specific intervention strategies is a detailed knowledge of the allergen sensitization profiles and the identification of the clinically most relevant allergens in the country. Using allergen extracts and recently defined allergen molecules rapid progress has already been made regarding the identification of important allergens in China. These studies have indicated that due to several factors including especially the occurrence of different climates in different regions of the country, heterogeneities regarding IgE sensitizations due to locally different exposomes exist in China. Due to the existence of cross-reactive allergens in different allergen sources, molecular IgE diagnosis is therefore required for the further deconvolution of IgE sensitization profiles to identify the clinically most important allergens in different regions of China and to develop allergen-specific forms of treatment and prevention.

## Author Contributions

RV and ND’s wrote the manuscript. RV, ND’s, and MW designed the figures and tables. ND’s, MW, MS, ES, and MF-T contributed materials. MF-T, ES, MW, SV, MC, MS, ND’s, and RV critically read and revised the manuscript. All authors contributed to the article and approved the submitted version.

## Funding

Supported by the Danube Allergy Research Cluster funded by the Country of Lower Austria, by the MCCA PhD program of the Austrian Science Fund (FWF), by the Russian Academic Excellence Project 5-100, by a Megagrant of the Government of the Russian Federation, grant No 14.W03.31.0024, by a research grant from Worg Pharmaceuticals, Hangzhou, China and by grant from HVD Life Science, Vienna, Austria. The funders were not involved in the study design, collection, analysis, interpretation of data, the writing of this article or the decision to submit it for publication.

## Conflict of Interest

RV has received research grants from HVD Life Science, Vienna Austria, Viravaxx, Vienna, Austria and Worg Pharmaceuticals, Hangzhou, China and serves as a consultant for Viravaxx and Worg. YL and RJ are employees of Worg Pharmaceuticals.

The remaining authors declare that the research was conducted in the absence of any commercial or financial relationships that could be construed as a potential conflict of interest.

## Publisher’s Note

All claims expressed in this article are solely those of the authors and do not necessarily represent those of their affiliated organizations, or those of the publisher, the editors and the reviewers. Any product that may be evaluated in this article, or claim that may be made by its manufacturer, is not guaranteed or endorsed by the publisher.

## Abbreviations

IgE, Immunoglobulin E; sIgE, specific IgE; IgG, Immunoglobulin G; IU, International Unit; ISU, ISAC standardized unit; ISAC, Immuno-Solid phase Allergy Chip; ELISA, Enzyme-linked immunosorbent assays; ABPA, Bronchopulmonary aspergillosis; Af, *Aspergillus fumigatus;* ALA, alpha lactalbumin; BLG, beta lactoglobulin; CAS, casein; SPT, skin prick test; PR10, pathogenesis-related protein family 10; LTP, lipid transfer protein; GST, glutathione S transferase; CCD, cross-reactive carbohydrate determinants; Bla g, *Blatella germanica*; Amb a, *Ambrosia artemisifolia*; AIT, allergen-specific immunotherapy; HDM, house dust mite; Der p, *Dermatophagoides pteronyssinus;* Blo t*, Blomia tropicalis;* Pen m, *Penaeus monodon*; Cha f, *Charybdis feriatus;* Ara h, *Arachis hypogaea;* Art v, *Artemisia vulgaris;* Phl p, *Phleum pratense*; Cyn d, *Cynodon dactylon*; SCIT, Subcutaneous ImmunoTherapy; CRD, Component Resolved Diagnosis; SPT, Skin Prick Test.
